# Molecular cancer prevention: Intercepting disease

**DOI:** 10.1002/1878-0261.70258

**Published:** 2026-04-18

**Authors:** Charlotte Grieco, Tej Pandya, Charles Swanton

**Affiliations:** ^1^ Cancer Evolution and Genome Instability Laboratory The Francis Crick Institute London UK; ^2^ Cancer Research UK Lung Cancer Centre of Excellence University College London Cancer Institute UK; ^3^ UKRI UCL Centre for Doctoral Training in AI‐Enabled Healthcare Systems University College London UK; ^4^ Department of Oncology University College London Hospitals UK

**Keywords:** cancer interception, chemoprevention, clinical trial design, inflammation, premalignant biomarkers, tumour initiation

## Abstract

Over several decades, therapeutic advances have transformed oncology, yet for many tumour types, survival improvements have been incremental, with substantial treatment‐related morbidity. A decisive pivot in oncology, from treating established malignancy to intercepting and preventing carcinogenesis, could deliver far greater population impact. Delivering this shift requires further mechanistic understanding of tumour initiation, validated biomarkers of premalignant progression and redesigned prevention trials in at‐risk populations. Regulatory and commercial frameworks must evolve to enable scalable molecular prevention. Such trials must deliver tolerable side effect profiles and rely on biologically validated surrogate endpoints rather than traditional survival outcomes. Cancer interception should be established as a core pillar of oncological management, alongside early detection and the therapeutic management of established disease, together creating an opportunity to reduce global disease burden at a scale that decades of therapeutic progress in advanced cancer have yet to achieve.

## Graphical abstract

Oncological practice must evolve, from treating established tumours to proactive cancer interception before clinical manifestation. This will require mechanistic insight into tumour initiation, validated biomarkers of early disease development and redesigned clinical trials, enabling cancer interception to become a core pillar of oncology with the potential to substantially reduce global disease burden.

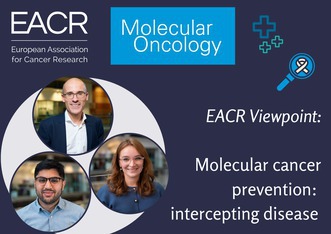

**Abbreviations**
CANOPY‐A: Canakinumab as Adjuvant Therapy in Adult Subjects with Completely Resected Non‐Small Cell Lung Cancer
CANTOS: Canakinumab Anti‐Inflammatory Thrombosis Outcomes Study
DNA: Deoxyribonucleic Acid
HPV: Human Papillomavirus
IL‐1β: Interleukin‐1 Beta
LDCT: Low‐Dose Computed Tomography
WHO: World Health Organization

## 1 Moving upstream: From cancer evolution to initiation

Cancer has historically been conceptualised as a mutational disease, exemplified by Knudson's two‐hit hypothesis [1]. Yet, through work demonstrating the prevalence of oncogenic mutations in histologically normal tissue and evidence that many carcinogens do not cause mutagenesis, it is clear that somatic mutations may be necessary but not sufficient for cancer initiation [2, 3, 4].

**Fig. 1:**
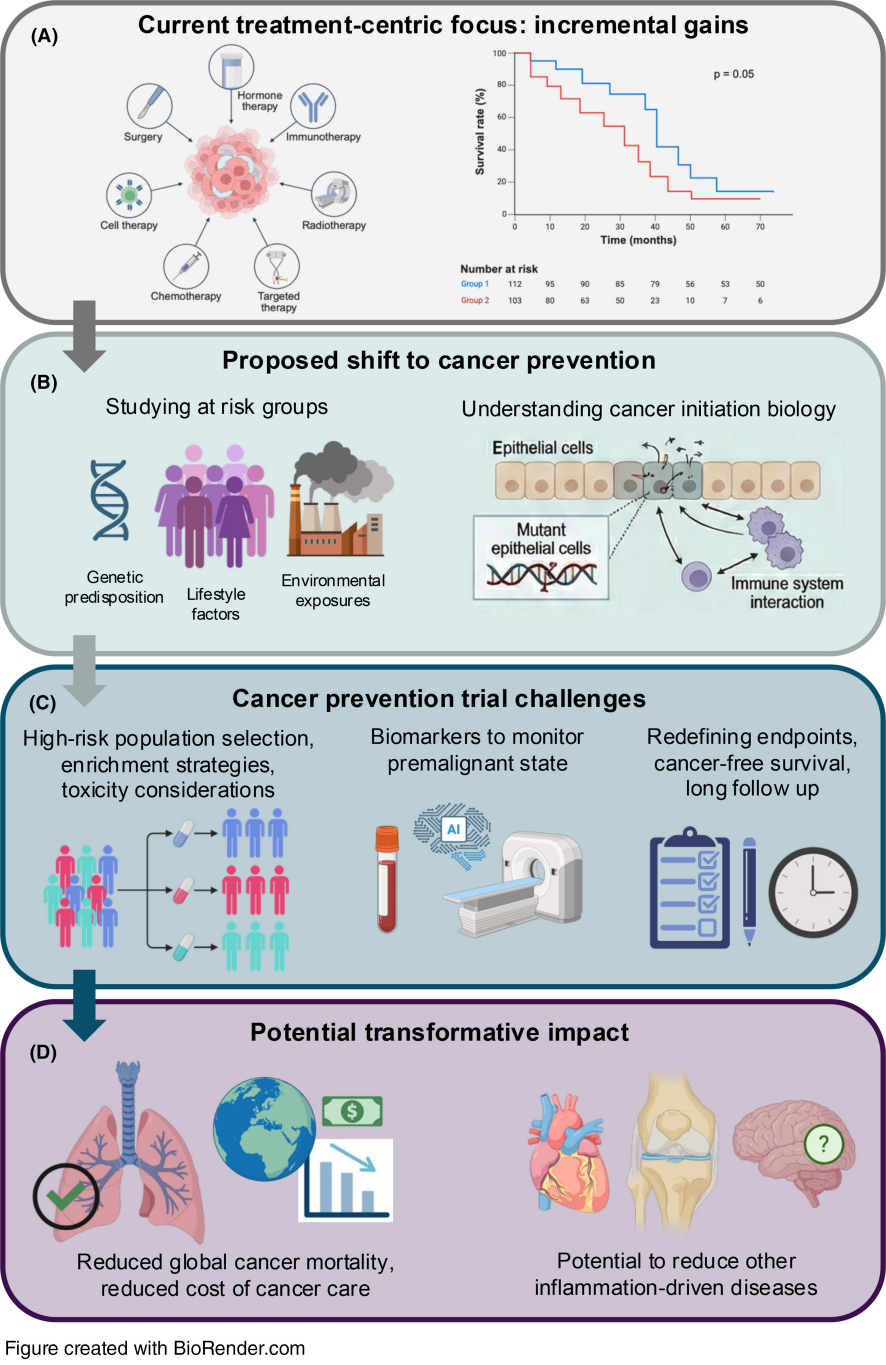
Conceptual framework for cancer prevention through early disease interception. (A) Current cancer management remains treatment‐centric, with therapies such as surgery, chemotherapy, immunotherapy, and targeted therapy yielding only incremental survival gains. (B) A proposed paradigm shift toward cancer prevention involves studying at‐risk groups defined by genetic predisposition, lifestyle factors, and environmental exposures, alongside a deeper understanding of cancer initiation biology, including mutant epithelial cell emergence and immune system interactions. (C) Key challenges for cancer prevention trials include high‐risk population selection and enrichment strategies, toxicity considerations, requirement of novel biomarkers to monitor the premalignant state, and the need to redefine endpoints around cancer‐free survival with prolonged follow‐up. (D) Successful implementation of this strategy holds transformative potential, including reduced global cancer mortality, lower costs of cancer care, and possible benefit for other inflammation‐driven diseases. Created in BioRender. Grieco, C. (2026) https://BioRender.com/7ht8y7q.

Since their inception over 25 years ago, Hanahan's latest update to the ‘Hallmarks of Cancer’ demonstrates our ever‐increasing understanding of the complexity of tumour‐host interactions [5]. Carcinogenesis is increasingly understood as a dynamic evolutionary process shaped by epithelial clonal competition, immune surveillance, stromal interactions and chronic inflammatory signalling. Established tumours represent heterogeneous Darwinian ecosystems, dynamically adapting both intrinsically and through reciprocal host interactions to persist despite treatment. The complexity of tumour‐host interactions and the number of escape mechanisms that evolve before treatment commencement mean true cures for established malignancy remain hard to achieve (Fig. 1A). Global cancer incidence continues to rise, largely driven by ageing populations, but in selected tumour types, through increasing incidence among younger adults [6]. These trends underscore the limitations of a treatment‐centric paradigm and highlight the need to interrogate earlier biological stages of disease development.

Evidence suggests that the process of cancer initiation is protracted, spanning years to decades, driven by the interplay of somatic evolution with immune surveillance mechanisms, both shaped by cumulative environmental exposures [7, 8]. This extended preclinical phase represents a biologically tractable window for therapeutic intervention. The success of vaccination against human papillomavirus (HPV) in reducing cervical cancer incidence illustrates what is possible when mechanistic causality, identifiable precursor lesions and effective intervention converge. Cervical cancer is now on a trajectory towards eradication, with the World Health Organization (WHO) targeting this milestone by 2030 [9]. While the biology of cancer initiation is less well defined in other tumour types, this case provides a blueprint for the key factors necessary to achieve similar success in molecular cancer prevention.

## 2 Identifying at‐risk groups

Non‐communicable diseases, including cardiovascular disease and cancer, remain leading causes of global mortality. Age‐adjusted mortality from cardiovascular disease has declined by nearly 75% in the last 50 years, driven by systematic risk factor modification and the broad deployment of preventive therapies targeting established biological drivers of the disease [10]. Reductions in cancer mortality have been comparatively modest. If age‐specific mortality rates remain stable, global cancer deaths are projected to rise by 63.7% by 2040 [6]. Investment in oncology has largely focused on established malignancy, with treatments such as increasingly sophisticated cellular therapies, becoming ever more complex, costly and frequently associated with substantial morbidity.

Well‐established risk factors, such as age and smoking, are already informing screening strategies in defined high‐risk populations, such as low‐dose computed tomography (LDCT) screening for lung cancer, which is being adopted in many countries worldwide [11]. Yet for other tumour types, aetiological drivers remain incompletely characterised. In addition, evolving epidemiological patterns, including rising incidence among younger adults, suggest that current risk models may be insufficient [6]. Modelling studies estimate that nearly half of lung cancers occur in patients not eligible for LDCT screening [12]. A more comprehensive and mechanistic understanding of cancer susceptibility across diverse populations is therefore essential to avoid under‐recognition of emerging at‐risk groups and should be a key focus of cancer prevention research across tumour types.

## 3 Deciphering premalignant trajectories

Effective molecular interception of carcinogenesis depends on a detailed characterisation of the trajectory from initial risk exposure to malignant transformation. However, profound heterogeneity exists both within and across tumour types, with premalignant states and evolutionary pathways shaped by tissue‐specific environmental and endogenous exposures (Fig. 1B). This variability underscores the need for cross‐tumour research to more precisely characterise these processes. Screening cohorts and large‐scale prospective resources such as the UK Biobank provide unique opportunities to characterise premalignant evolution [13]. Multi‐modal datasets incorporating imaging, genomics, proteomics and clinical variables may provide insight into the biological trajectory from exposure to malignant transformation, which can then be mechanistically interrogated in laboratories in genetically engineered model systems.

## 4 Divergent diseases may share common initiation pathways

An expanding body of biological evidence indicates that tumour promotion is a dynamic, context‐dependent process driven by evolving interactions between premalignant epithelial cells and the surrounding immune microenvironment [8]. Exploratory analysis of longitudinal data from the CANTOS trial, which evaluated canakinumab (anti‐IL‐1β) for secondary prevention of cardiovascular disease, revealed a dose‐dependent reduction in incident lung cancer, indicating that IL‐1β‐mediated inflammation may be a modifiable driver of cancer initiation [14]. However, the subsequent CANOPY‐A trial found no improvement in disease‐free survival when canakinumab was used as an adjuvant therapy in established lung cancer [15]. This divergence highlights a critical principle: biological pathways driving initiation may be distinct and not remain actionable once cancer is established. Timing is therefore fundamental to successful interception.

While an apparent distinction exists between tumour promotion and established cancer, there may be commonalities in the pathogenesis of other chronic inflammatory diseases. In CANTOS, the canakinumab‐treated group had reduced cardiovascular events and required fewer interventions for osteoarthritis [16]. Intercepting cancer initiation could confer broader benefits across inflammation‐driven diseases (Fig. 1D).

## 5 Monitoring the pre‐cancer milieu

Biomarkers capable of identifying tumour‐promoting states prior to overt malignancy are essential for molecular prevention. Current cell‐free DNA assays are optimised for detecting established cancer or minimal residual disease and therefore have limited utility in identifying pre‐cancer at‐risk states. A clinically useful biomarker for identifying individuals at risk of malignancy would need to capture the dynamic interplay between emerging premalignant clones and host immune surveillance. Plasma proteomics, a cell‐agnostic biomarker, may offer the potential to capture this evolving immune‐epithelial crosstalk.

In smoking‐related lung cancer, plasma proteomic analyses have revealed alterations in specific circulating proteins preceding cancer diagnoses, highlighting the potential of this approach to signal tumour initiation [17]. Elucidating the biological functions of these proteins may clarify mechanisms underlying cancer initiation, while longitudinal quantitative proteomic profiling could identify biologically actionable windows for interception. Integrating proteomics with other data modalities (e.g. radiological data) has the potential to enhance accuracy of risk stratification and longitudinal disease modelling. Artificial intelligence approaches can enhance composite biomarker models by optimising feature integration and enabling robust validation of predictive performance across large, independent cohorts. These biomarkers must be rigorously validated in prospective cohorts to ensure adequate sensitivity and specificity before safe use in guiding chemoprevention trials [18].

## 6 Cancer prevention trials: A paradigm shift

Translating molecular prevention concepts into clinical chemoprevention success presents challenges distinct from therapeutic trials in established cancer (Fig. 1C). Attempts at cancer prevention have typically required large cohorts and long follow‐up windows to achieve sufficient power for standard survival endpoints. Studies of this scale are expensive, making cancer prevention commercially unattractive to industry, in comparison to trials in diseases with faster survival endpoints. Prevention trials operate under fundamentally different ethical and safety constraints than therapeutic oncology studies. Participants are cancer‐free, and the absolute risk of disease may be modest; therefore, tolerance for toxicity is extremely low. Interventions must demonstrate not only efficacy but a highly favourable risk–benefit ratio when administered at scale and, potentially over extended durations [19].

Additionally, maintaining long‐term patient adherence is challenging when perceived immediate benefit is limited. Addressing the structural barriers to prevention trials requires deliberate redesign. Overall survival is an impractical primary endpoint in prevention settings, and even cancer incidence endpoints may necessitate prolonged and costly trials. Validated surrogate endpoints and biologically grounded biomarkers must therefore be developed in parallel with therapeutic strategies [20]. Cost and duration may be reduced through pragmatic, patient‐centred approaches such as remote follow‐up, streamlined trial procedures, and intermittent or limited‐duration dosing. Regulatory frameworks will also need to evolve to recognise these alternative endpoints.

## 7 Conclusion

Reorientating cancer research from established malignancy to tumour initiation represents a fundamental paradigm shift. Unlike early detection, where clonal evolution may already constrain reversibility, interception at the earliest stages of carcinogenesis offers a genuine opportunity to alter disease trajectory before malignant competence is acquired. Progress will depend on development of tools to identify premalignant evolution, utilising artificial intelligence tools for integration of multi‐modal data, and redefined clinical trial endpoints aligned to biological interception rather than post‐diagnosis survival. A sustained focus on cancer initiation and interception could achieve far greater population‐level reductions in mortality and simultaneously mitigate other inflammation‐driven diseases, achieving an impact comparable to the transformative successes of cardiovascular risk modification.

## Conflict of interest

TP has acted as a consultant for FutureHouse, Edison Scientific and Outlier AI; and is listed as an inventor (alongside CS) on patents filed in the UK related to risk prediction and early detection of lung cancer that have been filed but are currently unpublished and remain within the priority year. CS acknowledges grant support from AstraZeneca, Boehringer‐Ingelheim, Bristol Myers Squibb, Pfizer, Invitae (previously Archer Dx Inc—collaboration in minimal residual disease sequencing technologies), Ono Pharmaceutical, and Personalis. He is also Co‐Chief Investigator of the NHS Galleri trial funded by GRAIL and a paid member of GRAIL's Scientific Advisory Board. He was Chief Investigator for the AZ MeRmaiD 1 and 2 clinical trials and the Steering Committee Chair. CS is a paid board member for Novartis from March 2026. He is also a paid board member for Bicycle Therapeutics and is Chair of the Clinical Advisory Group. He receives consultant fees from Genentech, Medicxi, China Innovation Centre of Roche (CICoR) formerly Roche Innovation Centre – Shanghai, Relay Therapeutics (SAB member), Saga Diagnostics (SAB member), and Sarah Cannon Research Institute. He previously received consultant fees from Achilles Therapeutics. CS has received honoraria from Amgen, AstraZeneca, Bristol Myers Squibb, GlaxoSmithKline, Illumina, MSD, Novartis and Pfizer. CS has equity in Bicycle Therapeutics. He has stock options in Novartis, Relay Therapeutics, Saga Diagnostics and Bicycle Therapeutics. He has previously held stock and was co‐founder of Achilles Therapeutics. CS declares a patent application for methods to lung cancer (PCT/US2017/028013); targeting neoantigens (PCT/EP2016/059401); identifying patient response to immune checkpoint blockade (PCT/EP2016/071471); methods for lung cancer detection (US20190106751A1); identifying patients who respond to cancer treatment (PCT/GB2018/051912); determining HLA LOH (PCT/GB2018/052004); predicting survival rates of patients with cancer (PCT/GB2020/050221); methods and systems for tumour monitoring (PCT/EP2022/077987); analysis of HLA alleles transcriptional deregulation (PCT/EP2023/059039); relating to the use of plasma proteomics for risk prediction of lung cancer (PCT/EP2025/086701). CS is an inventor on a European patent application (PCT/GB2017/053289) relating to assay technology to detect tumour recurrence. This patent has been licensed to a commercial entity and under their terms of employment CS is due a revenue share of any revenue generated from such licence(s).

## Author contributions

CG conducted the literature review, wrote the manuscript and generated the graphical abstract. TP contributed to critical review and revision of the manuscript. CS conceived the article and provided supervision.
